# Response assessment of patients with locally advanced renal cell carcinoma receiving prior systemic therapy

**DOI:** 10.1186/s12885-026-15581-7

**Published:** 2026-01-16

**Authors:** Konstantin E. Seifert, Hendrik Dinkel, Linda Huberth, Dorothee Tiedje, Jan Gröticke, Laura-Maria Krabbe, Frederike Tepel, Kambiz Rahbar, Christof Bernemann, Andres J. Schrader, Martin Bögemann, Martin Janssen, Katrin Schlack, Barbara Heitplatz

**Affiliations:** 1 Department of Urology, University hospital Münster, Münster, Germany; 2 Department of Urology, Vivantes Network for Health, Berlin, Germany; 3 Department of Nuclear Medicine, University hospital Münster, Münster, Germany; 4 Gerhard-Domagk-Institute of Pathology, University hospital Münster, Münster, Germany

**Keywords:** Renal cell carcinoma, Immunotherapy, Nephrectomy, Tumor thrombus, Response assessment

## Abstract

**Background:**

Locally advanced renal cell carcinoma (RCC) without distant metastases remains challenging to treat surgically, especially when venous tumor thrombus is present. Neoadjuvant checkpoint inhibitor (CPI) and tyrosine kinase inhibitor (TKI) therapy may downstage tumors and improve resectability, but evidence is limited. This study evaluated radiographic and pathological responses, surgical feasibility, and safety following preoperative systemic therapy.

**Methods:**

This single-center retrospective analysis of 17 patients with non-metastatic RCC who received ≥ 1 month of CPI, TKI, or CPI + TKI before nephrectomy. Tumor stage, radiographic tumor/thrombus reduction, pathological viable tumor percentage, surgical outcomes, complications by Clavien-Dindo, and disease-free survival (DFS) were collected.

**Results:**

Median pretreatment tumor diameter was 9.1 cm; 71% had venous tumor thrombus. Most patients (88%) received CPI + TKI. Median treatment duration was 7 months. Median radiographic tumor reduction was 27.1%; 50% of thrombus-bearing patients showed Mayo level reduction. Median viable tumor content was 30%; 17.6% achieved ypT0. T-stage downstaging occurred in 53%. Surgery was feasible in all cases; 17.6% experienced Clavien–Dindo grade ≥ III complications, mostly with thrombus resections. Median DFS was 28 months. No correlation between radiographic and pathological response was observed. Limitations include retrospective single-center design, small sample size, and limited follow-up/events, restricting generalizability and survival analyses.

**Conclusion:**

Preoperative CPI + TKI therapy in selected non-metastatic RCC patients is associated with measurable tumor and thrombus regression without compromising surgical safety. These findings support further prospective trials to clarify long-term oncologic benefit and identify predictors of response to integrate neoadjuvant therapy into multidisciplinary RCC care.

## Introduction

Renal cell carcinoma (RCC) is a common cancer in adults with approximately 400,000 new cases diagnosed worldwide in 2020. Even though a rising number of cases is observed, mainly due to better diagnostic approaches and the possibility of local approaches at an early stage, the mortality rate has declined [1]. Live prolonging agents like check-point inhibitors (CPI) and tyrosine-kinase inhibitors (TKI) might have an additional impact on the declined mortality rate as well [2].

For patients with non-metastatic renal cell carcinoma, the favored treatment approach remains either kidney-sparing surgery or radical nephrectomy [3]. To date, only a few patients are eligible for adjuvant therapy following surgical intervention. The Keynote-564 trial is the only study that has shown a benefit in disease-free survival (DFS) and overall survival (OS) with 12-months of pembrolizumab compared to placebo in patients with locally advanced high-risk RCC following surgery [4]. Other adjuvant or perioperative trials failed to show a significant benefit [5–7]. Regarding neoadjuvant CPI treatment in RCC, no randomized prospective data are currently available, but some trials are still ongoing [8]. This also applies to TKI monotherapy trials which at least demonstrated meaningful tumor downstaging [9–11].

Prospective phase II trials evaluating the primary tumor response following neoadjuvant CPI plus TKI therapy reported a tumor size reduction of 13–20% and a median of residual viable tumor cells of 50% [12, 13]. In contrast, a neoadjuvant trial evaluating four cycles of nivolumab monotherapy prior to surgery only led to a primary tumor reduction of approximately 1%, supporting the need to add a TKI in the neoadjuvant setting for relevant tumor shrinkage [14]. In a retrospective multicenter analysis of 52 patients with advanced or metastatic RCC with prior CPI or CPI plus TKI therapy, a downstaging in T-stage was observed in nearly half of the patients [15]. Furthermore, two prospective trials evaluated the response of the venous tumor thrombus after neoadjuvant therapy with either TKI monotherapy or TKI plus CPI combination therapy [16, 17]. In both trials, a reduction of the venous tumor thrombus according to the Mayo classification was achieved.

Collectively, these neoadjuvant trials showed promising results in terms of tumor or thrombus size reduction, thus improving surgical feasibility.

The aim of this retrospective analysis was to evaluate the pathological and radiographic response in patients with advanced RCC who underwent induction therapy, as well as to assess the safety and feasibility of surgical resection following preoperative systemic therapy.

## Materials and methods

### Patients

In this single center retrospective study, we evaluated patients treated between July 2019 and February 2025 who received systemic treatment prior to surgery for locally advanced renal cell carcinoma at the Department of Urology, University Hospital Muenster, Germany. All cases were reviewed by an interdisciplinary tumor board, and treatment was initiated after obtaining written informed consent. Prior systemic treatment was administered if patients were not suitable for immediate surgical intervention or if immediate surgery was not preferred. Immediate surgical intervention was not suitable in cases with large primary tumors or venous tumor thrombus extension. All patients underwent tumor biopsy prior to initiating systemic treatment. Not all patients were initially planned for a deferred surgical approach but were referred for surgery due to an objective response. In most cases, surgery was planned when no further tumor reduction was observed. Patients with distant metastases were excluded. Eligible systemic treatments were either TKI, TKI + CPI, or CPI + CPI, and each treatment was administered for at least one month prior to undergoing partial or radical nephrectomy. Ethics committee-approval was granted (Ethikkomission der Aerztekammer Westfalen-Lippe und der Medizinischen Fakultaet der Universitaet Muenster: 2025-101-f-S). This study was conducted in accordance with the Declaration of Helsinki (as revised in 2013).

### Data collection

Patients received therapy at regular intervals, accompanied by continuous clinical and radiological assessments. Digital patient data were retrieved from the hospital information system (ORBIS^®^, Dedalus HealthCare GmbH, Bonn, Germany).

Patient-related demographic data were recorded, including age, sex, disease progression, and death. The clinical tumor stage was documented at the start of therapy. Tumor staging at baseline until surgery and surgical details were documented.

Pathological grading, pathological tumor stage, and histological classification were recorded. Postoperative complications were assessed using the Clavien-Dindo classification [18].

### Pathological response assessment

The surgical specimen was received in 4% buffered formalin and fixed for 24 h. Gross examination included description of the tumor, measurement of its size, and assessment of the distances to the resection margins. Representative sections of the tumor were embedded, including adjacent tissue and resection margins. Histopathological evaluation was performed on hematoxylin and eosin-stained slides, with reporting of viable residual tumor.

### Radiographic response assessment

Patients underwent either computed tomography or magnetic resonance imaging. Radiographic evaluation included the measurement of the primary tumor size (largest diameter in cm) during staging and the percentage of primary tumor reduction over time.

In case a vena cava tumor thrombus was present, the response was measured by comparing the change in thrombus length (in cm) between baseline and preoperative imaging and by evaluating Mayo thrombus level changes [19].

### Statistical analysis

Statistical assessment was performed using SPSS-Statistics V25.0 (IBM Inc., Armonk, NY) and Prism 10 V10.4.0 (GraphPad Software, LLC., San Diego, CA). Descriptive statistics are reported as medians with interquartile ranges (IQR) or means with 95% confidence intervals (CI) for continuous variables, unless otherwise stated. Statistical significance was set at *p* < 0.05. The Pearson correlation coefficient was used to assess the linear relationships between continuous variables. Kaplan-Meier analysis was used to estimate DFS.

## Results

### Patient characteristics

This study included 17 patients with RCC who underwent systemic therapy followed by surgical resection. The mean age at diagnosis was 65 years (95%CI: 62–68), with 12 male and 5 female patients. Most patients presented with clear cell histology (94.1%), while one patient had papillary RCC. No patient presented with sarcomatoid features. The mean size of the primary renal tumor in largest diameter, as assessed by imaging prior to initiation of systemic therapy, was 9.1 cm (95%CI: 7.1–11.2). Based on imaging, the clinical T-stage at diagnosis was cT2 in three patients (17.7%), cT3 in 13 (76.5%), and cT4 in one (5.9%). The cT3a, cT3b, and cT3c stage were 11.8%, 52.9% and 11.8%, respectively. Three patients had suspected lymph node metastases. No distant metastases were present in any patient at the initiation of systemic treatment. The patient characteristics are summarized in Table 1.

**Table 1:** Patient characteristics

	Patients (*n* = 17)
Age, years	
Median (IQR)	64 (62–70)
Sex	
Male	12 (70.6%)
Female	5 (29.4%)
Histological subtype	
Clear cell carcinoma	16 (94.1%)
Papillary carcinoma	1 (5.9%)
Initial tumor size in cm	
Median	8.6 (5.9–10.6)
cT-stage	
cT2	3 (17.7%)
cT3a	2 (11.8%)
cT3b	9 (52.9%)
cT3c	2 (11.8%)
cT4	1 (5.9%)
Systemic treatments	
Ipilimumab/Nivolumab	1 (5.9%)
Cabozantinib/Nivolumab	1 (5.9%)
Lenvatinib/Pembrolizumab	13 (76.5%)
Cabozantinib	2 (11.8%)
Time to surgery in month	
Median	7 (3.5-9)
Venous tumor thrombus	
No	5 (29.4%)
Yes	12 (70.6%)
Surgical approach	
Open	14 (82.4%)
Robotic assisted	3 (17.6%)
Type of nephrectomy	
Partial	3 (17.6%)
Radical	14 (82.4%)

Fifteen patients (88.2%) received combination therapy and two patients (11.8%) cabozantinib monotherapy. Combination therapies included ipilimumab plus nivolumab (1 patient (5.9%)), lenvatinib plus pembrolizumab (13 patients (76.5%)), and cabozantinib plus nivolumab (1 patient (5.9%)). The median duration of systemic treatment prior to surgery was 7 months (95%CI: 5–8). Fourteen (82.4%) patients underwent radical nephrectomy, whereas only three underwent partial nephrectomy. An open surgical approach was chosen in 14 (82.4%) patients. A venous tumor thrombus was present in 12 patients (70.6%) and was successfully removed all cases. The mean length of the venous tumor thrombus was 8 cm (95%CI: 5.3–10.6). The median Mayo level was 2 (Range: 1–4).

### Pathological response

**Fig. 1:**
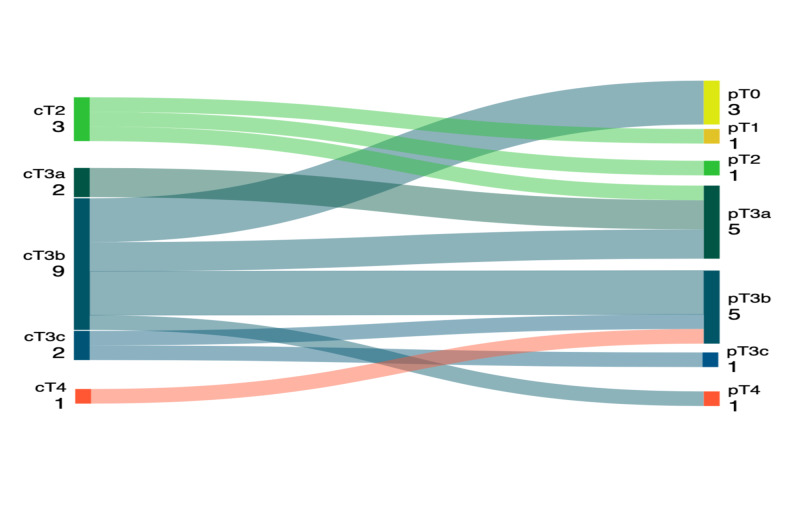
Sankey diagram of the T-stage migration from initial staging to pathological outcome

**Fig. 2:**
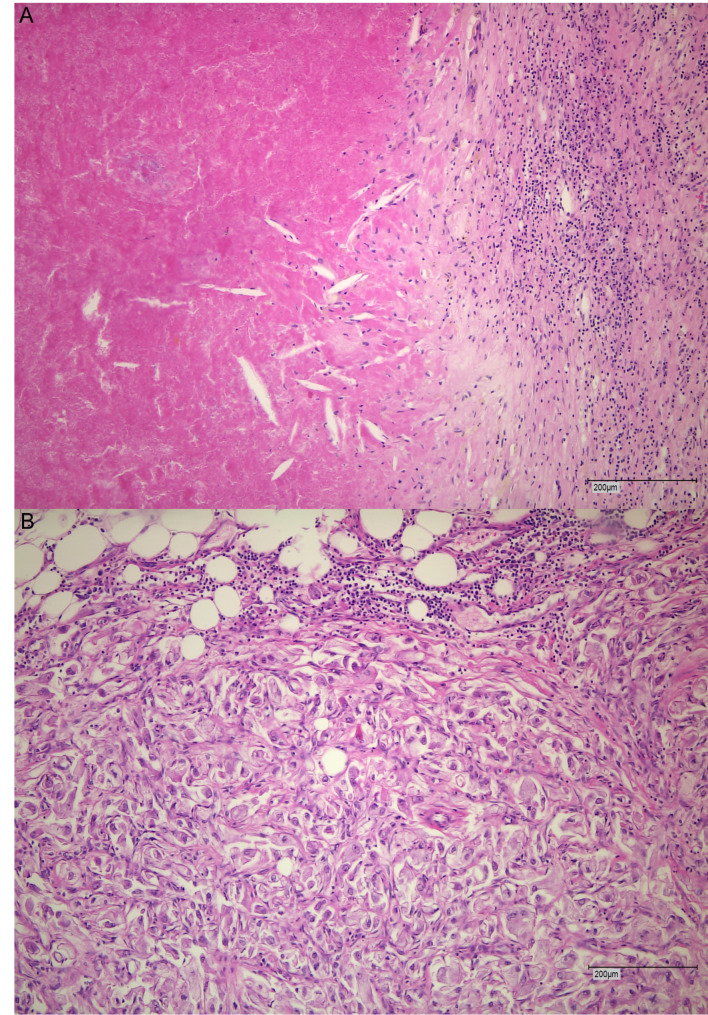
Hematoxylin and eosin-stained slides representing 0% viable tumor cells (**A**) and 90% viable tumor cells (**B**). 100x magnification

After preoperative treatment, the median percentage of viable tumor cells in the specimens was 30% (IQR: 15–70). Three patients (17.6%) had no evidence of viable tumor cells and were classified as ypT0. Each of the stages ypT1, ypT2, and ypT4 was present in one patient (5.9%), and 11 patients (64,7%) had a ypT3 stage. The ypT3a, ypT3b, and ypT3c stages were found in 29.4%, 29.4% and 5.9% of cases, respectively. Figure 1 shows the T-stage migration in the patient cohort. Downstaging occurred in nine patients (52.9%) whereas two patients (11.8%) showed greater extent of expected disease burden according to previous radiological staging. Six patients (35.3%) had a stable disease in terms of T stage. In one case, lymph nodal involvement was confirmed pathologically. Adrenal metastasis was observed in one patient. Histopathological images representing 0% and 90% viable tumor cells are shown in Fig. 2.

### Radiographic response

**Fig. 3:**
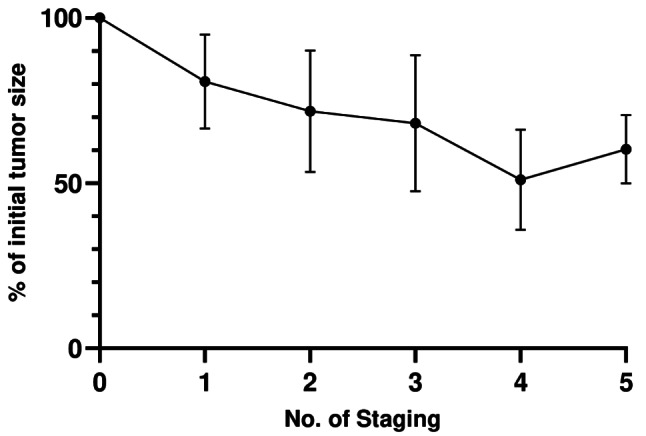
Percentage of change over time of all tumors from initiation of systemic treatment until surgery, stratified by number of stagings performed

The overall response rate of the primary tumor was 35.3%, with six patients showing a partial response, 11 patients with stable disease, and no patients with progressive disease. The median radiographic reduction was 27.1% (IQR: 9.5–44.8). In the 14 patients receiving CPI plus TKI the median reduction was slightly higher with 29.1% (IQR: 10.8–47.6). An overview of the radiographic as well as pathological response between the different treatment groups is shown in Table 2. Of the 12 patients with a venous tumor thrombus, 6 patients (50%) had a reduction in the Mayo level. The percentage of change was in median 21.2% (IQR: 4.2–37.5) to a mean size of 5.7 cm (95%CI: 3.7–7.8). The time from initiation of therapy to surgery was significantly longer in the radiographic response group ≥ 27% compared to ≤ 27% group (8 vs. 4.5 months, *p* = 0.021). The percentage of change in all patients from the initial to the last staging is shown in Fig. 3. No correlation was observed between the radiographic response and percentage of viable tumor cells (Pearson’s correlation coefficient =-0.118; *p* = 0.653).

**Table 2:** Main radiological and pathological differences in each group (TKI-monotherapy vs. TKI + CPI). TKI = tyrosine-kinase inhibitors; TKI + CPI = tyrosine-kinase inhibitors + check-point inhibitors

Characteristics	TKI monotherapy (*n* = 2)	TKI + CPI (*n* = 14)
% of viable residual tumor		
Median (IQR)	35%	30% (7.5–72.5)
% primary tumor reduction		
Median (IQR)	5.4%	29.1% (19.8–47.6)
Initial Mayo level		
0	0	4 (28.6%)
1	0	1 (7.1%)
2	0	6 (42.9%)
3	1 (50%)	1 (7.1%)
4	1 (50%)	2 (14.3%)
Preoperative Mayo level		
0	0	5 (35.7%)
1	0	2 (14.3%)
2	1 (50%)	5 (35.7%)
3	1 (50%)	1 (7.1%)
4	0	1 (7.1%)
Mayo level reduction	1 (50%)	5 (50%)

### Surgical outcomes

The median operating time was 244 min (IQR: 167–277) with a significant difference between open and robotic-assisted surgery (median 255 vs. 140 min (*p* = 0.021)). No significant difference was observed between radical or partial nephrectomy or if a venous tumor thrombus was present (both *p* = 0.51). Postoperative complications were infrequent, with only three events (17.6%) classified as Clavien-Dindo grade III or higher. One patient died postoperatively, but this was most likely due to progressive disease and new undetected brain metastases. However, this patient was classified as having a Clavien-Dindo grade V complication. Six patients (35.3%) had no recorded complications, and 1 (5.9%) and 7 (41.2%) patients had grade I and II complications, respectively. Table 3 summarizes the complications observed postoperatively. All grade III or higher complications occurred in patients with venous tumor thrombus, thus more complex surgery.

**Table 3:** Postoperative complications observed. Patients could have had more than one complication

Complication	Number of patients
No complications	6 (35.3%)
Oral antibiotics	1 (5.9%)
i.v. antibiotics	1 (5.9%)
Erythrocyte transfusion	8 (47.1%)
Revision surgery	2 (11.8%)
Death	1 (5.9%)

### Disease-free survival

**Fig. 4:**
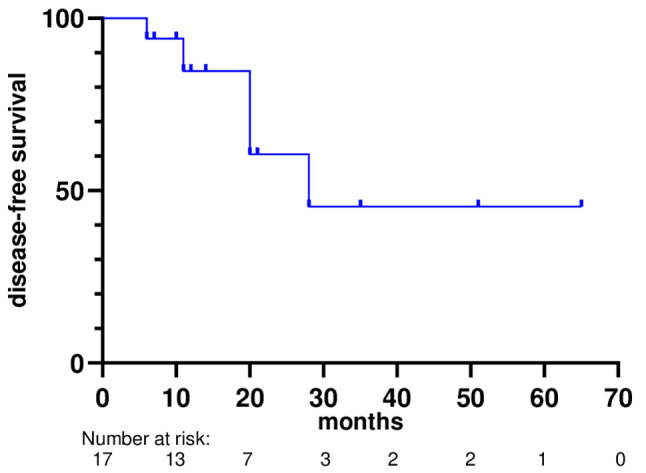
Kaplan-Meier analysis of the disease-free survival after initiation of systemic treatment

The median DFS was 28 months (95%CI: not reached), as shown in the Kaplan-Meier curve (Fig. 4). No significant differences in the DFS were observed between the groups. Only five events were recorded in this study.

## Discussion

In this retrospective single-center study, we evaluated the impact of induction systemic therapy on surgical outcomes, pathological response, and radiographic tumor burden in patients with locally advanced RCC without distant metastases. Our findings underscore the feasibility and safety of surgery following systemic pretreatment and suggest a meaningful pathological and radiographic tumor response in a subset of patients.

Consistent with prior studies, the combination of CPI and TKI appears to be associated with greater tumor shrinkage than monotherapy. Our cohort demonstrated a median radiographic tumor size reduction of 27.1%, with CPI + TKI combinations yielding a slightly higher median reduction of 29.1%. These outcomes are in line with recent phase II studies that reported primary tumor shrinkage rates of 13–20% for CPI + TKI combinations such as avelumab/axitinib and sitravatinib/nivolumab [12, 13]. In contrast, neoadjuvant CPI monotherapy (nivolumab) resulted in minimal radiographic tumor response (~ 1%), as shown by Carlo et al. [14]. The objective response rate (ORR) of 35.3% observed in our cohort is notably lower than the rates typically reported in metastatic RCC populations treated with TKI + CPI combinations, where responses exceed 60–70% [20, 21]. Several factors may account for this discrepancy. First, primary tumors may exhibit different sensitivity compared to metastatic lesions, potentially due to variations in tumor microenvironment, vascularization, and immune infiltration [22]. Second, our patient selection criteria, which required locally advanced disease without distant metastases, may have inadvertently enriched for tumors with intrinsic resistance to systemic therapy [23]. Importantly, despite the modest ORR, we observed substantial pathological responses with a median of 30% viable tumor cells and 52.9% of patients achieving pathological downstaging, highlighting a dissociation between radiographic and pathological response, a phenomenon previously shown in the NEOAVAX trial [24]. This discordance suggests that radiographic response alone may underestimate the true biological effect of systemic therapy on primary tumor burden. The 10% higher reduction of the primary tumor size in our cohort could be due to the longer systemic treatment duration of 7 months compared to 50 days in the trial of Karam et al. (sitravatinib/nivolumab). We also showed that prolonged prior systemic treatment was associated with a better radiographic primary tumor response. However, in our cohort, treatment was continued until disease progression, patient request, or absence of further tumor size reduction.In the NEOAVAX trial, the pathological response was not associated with the radiologic response [24]. These findings were also confirmed in our cohort. These findings raise the question of whether either of these parameters could be used as a predictor for survival.

Histopathologically, a median of 30% viable tumor cells was observed in the surgical specimens, and 17.6% of patients achieved a complete pathological response (pT0). This is less than that previously reported in most prospective neoadjuvant RCC studies, which showed a median of 50% viable tumor cells remaining post-treatment [14]. However, the percentage of viable tumor cells did not correlate with DFS or duration of systemic therapy. Thus, the significance of examining viable tumor cells remains unclear, and larger cohorts are needed. Importantly, more than half of the patients experienced T-stage downstaging, which is consistent with the observations made by Panian et al. They reported a T-stage downstaging in approximately 50% of patients undergoing surgery post-immunotherapy [15]. This degree of pathological response could translate into better long-term oncologic outcomes, although our study was not powered to evaluate this. T-stage downstaging correlates with the radiographic response, as the TNM classification mainly relies on tumor size and the extent of the venous tumor thrombus.

A distinct and clinically relevant observation was the response of venous tumor thrombus. Six out of twelve patients with vena cava involvement showed a reduction in Mayo level after therapy, and no patients showed an increase. This finding aligns with the results of the NAXIVA and NEOTAX studies, which demonstrated that neoadjuvant TKI or CPI + TKI therapy can significantly downstage vena cava tumor thrombus [16, 17]. Given the complexity and morbidity associated with thrombectomy, any reduction in thrombus size or level may facilitate surgical resection and reduce perioperative risk.

Surgical resection following systemic therapy was feasible in all cases, with acceptable complication rates reported. Most adverse events were of low grade, and only 17.6% of patients experienced Clavien-Dindo grade III or higher complications. These results compare favorably with those of prior surgical series involving high-risk or thrombus-bearing tumors [16, 17]. Importantly, the use of induction therapy did not delay surgery beyond the clinically relevant window. In fact, the group with a higher radiographic response tended to receive therapy for a longer period. While limited comparative data exist, neoadjuvant therapy offers theoretical advantages over adjuvant approaches by downsizing tumors and vena cava thrombi, reducing operative complexity and perioperative morbidity, and allowing pathological assessment of treatment response. Our observation that six of twelve patients with vena cava involvement demonstrated thrombus downstaging suggests that patients with extensive vena cava involvement may be optimal candidates for neoadjuvant therapy, as reduction in thrombus complexity can significantly decrease perioperative morbidity. However, prospective biomarker studies are needed to identify which patients are most likely to benefit from induction or neoadjuvant therapy, as current radiographic and pathological response assessments did not consistently predict long-term disease-free survival. In addition to that, there is no perioperative or neoadjuvant phase 3 trial that showed positive results in terms of disease-free or overall survival, whereas at least one adjuvant phase 3 trial led to the approval of pembrolizumab in this setting [4, 7].

Despite these promising findings, this study has some limitations. The retrospective design and small sample size limit the generalizability of our findings. Although a median disease-free survival of 28 months was observed, the follow-up duration and number of events were limited. In the NEOAVAX trial, the median DFS was 25.1 months, which is comparable with our results despite limited events [24]. Another limitation is that therapeutic heterogeneity represents a significant confounding factor. Our cohort included multiple systemic regimens (CPI + TKI combinations, CPI monotherapy, and TKI monotherapy), which may differentially affect efficacy and outcomes due to distinct pharmacokinetic profiles and toxicity patterns. This heterogeneity limits our ability to draw definitive conclusions about specific treatment regimens. The absence of a standardized methodology for reporting pathological response in RCC is another critical limitation. While we employed viable tumor cells, other groups have utilized different grading systems (e.g., percentage necrosis or degree of fibrosis), limiting direct comparability across studies and meta-analyses [25]. Treatment duration represents another confounding factor due to timing of surgical intervention. Patients with unfavorable early responses discontinued therapy earlier, while responders continued longer, making it difficult to distinguish whether treatment duration is a determinant or consequence of response. Larger retrospective analyses and randomized prospective studies are required to assess the long-term oncologic benefits of this approach. However, as the only randomized perioperative phase 3 trial was negative, other outcome measurements should be considered, such as surgical outcome measurements and downstaging.

Future studies should focus on validating radiographic and pathological markers that can predict response. Furthermore, randomized prospective trials are urgently needed to establish the role of neoadjuvant therapy in non-metastatic RCC. Ongoing phase II studies investigating CPI-based neoadjuvant regimens (e.g., NCT05733715, NCT05319015) will be important in defining optimal strategies and identifying subgroups most likely to benefit [8, 26].

In conclusion, our data support the use of prior CPI + TKI therapy in selected patients with locally advanced RCC, especially in cases where a venous tumor thrombus is present. This approach is associated with measurable radiographic and pathological tumor responses, a reduction in venous tumor thrombus in selected cases, and does not compromise surgical safety. These findings further support the integration of systemic therapy into multidisciplinary management of high-risk, non-metastatic RCC.

## Data Availability

The data that support the findings of this study are available from the corresponding author upon reasonable request.

## Abbreviations

CPI: Checkpoint inhibitor
DFS: Disease-free survival
ORR: Overall response rate
OS: Overall survival
RCC: Renal cell carcinoma
TKI: Tyrosine kinase inhibitor
